# *N*,*N*′-Bis(3-methylbut-2-enyl)-*N*,*N*′-(1,4-phenylene)dibenzenesulfonamide

**DOI:** 10.1107/S1600536811045661

**Published:** 2011-11-12

**Authors:** Ejaz, Islam Ullah Khan, Tahir Ali Sheikh, William T. A. Harrison

**Affiliations:** a Materials Chemistry Laboratry, Department of Chemistry, GC University, Lahore 54000, Pakistan; b Department of Chemistry, University of Aberdeen, Meston Walk, Aberdeen AB24 3UE, Scotland

## Abstract

The complete mol­ecule of the title compound, C_28_H_32_N_2_O_4_S_2_, is generated by a crystallographic inversion centre. The dihedral angle between the central and pendant aromatic rings is 46.78 (7)°. The C_ar_—S—N—C_ar_ (ar = aromatic) torsion angle is 73.64 (15)° and the bond-angle sum for the N atom is 350.4°. In the crystal, weak C—H⋯O inter­actions link the mol­ecules, forming a two-dimensional network lying parallel to the *bc* plane.

## Related literature

For related structures, see: Ejaz *et al.* (2011*a* ▶,*b* ▶).
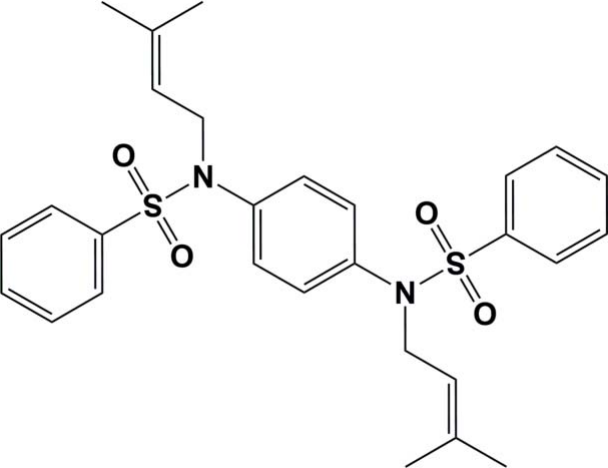


## Experimental

### 

#### Crystal data

C_28_H_32_N_2_O_4_S_2_
*M* *_r_* = 524.68
Monoclinic, 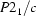

*a* = 10.6574 (4) Å
*b* = 20.8073 (8) Å
*c* = 6.4015 (2) Å
β = 105.673 (2)°
*V* = 1366.76 (8) Å^3^
*Z* = 2
Mo *K*α radiation
μ = 0.23 mm^−1^
*T* = 296 K
0.50 × 0.20 × 0.15 mm

#### Data collection

Bruker APEXII CCD diffractometer
11502 measured reflections
2667 independent reflections
1931 reflections with *I* > 2σ(*I*)
*R*_int_ = 0.031

#### Refinement

*R*[*F* ^2^ > 2σ(*F* ^2^)] = 0.039
*wR*(*F* ^2^) = 0.104
*S* = 1.03
2667 reflections
165 parameters
H-atom parameters constrained
Δρ_max_ = 0.23 e Å^−3^
Δρ_min_ = −0.31 e Å^−3^

### 

Data collection: *APEX2* (Bruker, 2007 ▶); cell refinement: *SAINT* (Bruker, 2007 ▶); data reduction: *SAINT*; program(s) used to solve structure: *SHELXS97* (Sheldrick, 2008 ▶); program(s) used to refine structure: *SHELXL97* (Sheldrick, 2008 ▶); molecular graphics: *ORTEP-3* (Farrugia, 1997 ▶); software used to prepare material for publication: *SHELXL97*.

## Supplementary Material

Crystal structure: contains datablock(s) I, global. DOI: 10.1107/S1600536811045661/su2338sup1.cif

Structure factors: contains datablock(s) I. DOI: 10.1107/S1600536811045661/su2338Isup2.hkl

Supplementary material file. DOI: 10.1107/S1600536811045661/su2338Isup3.cml

Additional supplementary materials:  crystallographic information; 3D view; checkCIF report

## Figures and Tables

**Table 1:** Hydrogen-bond geometry (Å, °)

*D*—H⋯*A*	*D*—H	H⋯*A*	*D*⋯*A*	*D*—H⋯*A*
C2—H2⋯O2^i^	0.93	2.52	3.398 (3)	158

Symmetry code: (i) 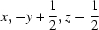.

**Fig. 1.**
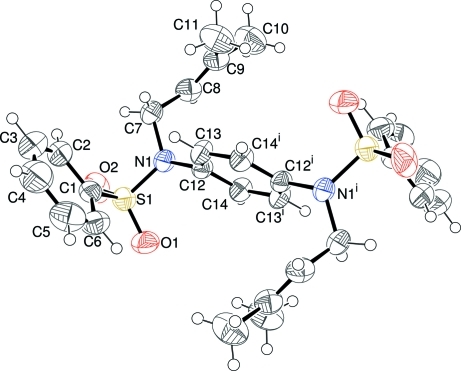
The molecular structure of the title molecule, showing 50% displacement ellipsoids and the atom numbering scheme [symmetry code: (i) -x+1, -y+1, -z+1].

## supplementary crystallographic information

### Comment

As part of our ongoing studies of symmetrical aryl sulfonamides (Ejaz *et al.*, 2011*a*,*b*), the synthesis and structure of the title compound are described herein.

The complete molecule of the title compound is generated by a crystallographic inversion centre (Fig. 1). The dihedral angles between the central benzene ring (C12-C17) and the pendant (C1-C6) ring is 46.78 (7)°. Overall, an approximante H-shaped conformation arises. The C1—S1—N1—C12 torsion angle is 73.64 (15)°, indicating a *gauche* conformation for the sulfonamide bridge between the aromatic rings. The bond angle sum of 350.4° for the nitrogen atom, N1, is similar to the situation found for two related structures, *N*,*N*'-(benzene-1,3-diyldimethanediyl)dibenzenesulfonamide and *N*,*N*'-diethyl-(benzene-1,3-diyldimethanediyl) dibenzenesulfonamide, that we have reported on recently (Ejaz *et al.*, 2011*a*,*b*).

In the crystal, weak C—H···O interactions link the molecules to form a two-dimensional network lieing parallel to the bc plane (Table 1). There is no aromatic π-π stacking in the crystal.

### Experimental

A mixture of *N*,*N*'-benzene-1,4-diyldibenzenesulfonamide (0.194 g, 0.5 mmol), sodium hydride (0.24 g; 1.0 mmol) and *N*,*N*-dimethylformamide (10.0 ml) was stirred in a 100-ml round-bottom flask at room temperature for half an hour followed by the addition of 3,3-dimethylallyl bromide (0.116 ml; 1.0 mmol). The reaction mixture was stirred for five hours; reaction progress was monitored by TLC. After completion, the contents were poured over crushed ice. The precipitated product was isolated, washed and crystallized from methanol to yield brown block-like crystals of the title compound.

### Refinement

The hydrogen atoms were placed in calculated positions (C—H = 0.93–0.96 Å) and refined as riding atoms with *U*_iso_(H) = 1.2*U*_eq_(C) or 1.5*U*_eq_(methyl C). The methyl groups were allowed to rotate, but not to tip, to best fit the electron density.

### Crystal data

**Table:**

C_28_H_32_N_2_O_4_S_2_	*F*(000) = 556
*M**_r_* = 524.68	*D*_x_ = 1.275 Mg m^−^^3^
Monoclinic, *P*2_1_/*c*	Mo *K*α radiation, λ = 0.71073 Å
Hall symbol: -P 2ybc	Cell parameters from 3403 reflections
*a* = 10.6574 (4) Å	θ = 2.0–28.4°
*b* = 20.8073 (8) Å	µ = 0.23 mm^−^^1^
*c* = 6.4015 (2) Å	*T* = 296 K
β = 105.673 (2)°	Block, brown
*V* = 1366.76 (8) Å^3^	0.50 × 0.20 × 0.15 mm
*Z* = 2	

### Data collection

**Table:**

Bruker APEXII CCD diffractometer	1931 reflections with *I* > 2σ(*I*)
Radiation source: fine-focus sealed tube	*R*_int_ = 0.031
graphite	θ_max_ = 26.0°, θ_min_ = 2.8°
ω scans	*h* = −13→13
11502 measured reflections	*k* = −25→25
2667 independent reflections	*l* = −7→7

### Refinement

**Table:**

Refinement on *F*^2^	Primary atom site location: structure-invariant direct methods
Least-squares matrix: full	Secondary atom site location: difference Fourier map
*R*[*F*^2^ > 2σ(*F*^2^)] = 0.039	Hydrogen site location: inferred from neighbouring sites
*wR*(*F*^2^) = 0.104	H-atom parameters constrained
*S* = 1.03	*w* = 1/[σ^2^(*F*_o_^2^) + (0.0426*P*)^2^ + 0.3607*P*] where *P* = (*F*_o_^2^ + 2*F*_c_^2^)/3
2667 reflections	(Δ/σ)_max_ = 0.001
165 parameters	Δρ_max_ = 0.23 e Å^−^^3^
0 restraints	Δρ_min_ = −0.31 e Å^−^^3^

### Special details

**Table:**

Geometry. All e.s.d.'s (except the e.s.d. in the dihedral angle between two l.s. planes) are estimated using the full covariance matrix. The cell e.s.d.'s are taken into account individually in the estimation of e.s.d.'s in distances, angles and torsion angles; correlations between e.s.d.'s in cell parameters are only used when they are defined by crystal symmetry. An approximate (isotropic) treatment of cell e.s.d.'s is used for estimating e.s.d.'s involving l.s. planes.
Refinement. Refinement of *F*^2^ against ALL reflections. The weighted *R*-factor *wR* and goodness of fit *S* are based on *F*^2^, conventional *R*-factors *R* are based on *F*, with *F* set to zero for negative *F*^2^. The threshold expression of *F*^2^ > σ(*F*^2^) is used only for calculating *R*-factors(gt) *etc*. and is not relevant to the choice of reflections for refinement. *R*-factors based on *F*^2^ are statistically about twice as large as those based on *F*, and *R*- factors based on ALL data will be even larger.

### Fractional atomic coordinates and isotropic or equivalent isotropic displacement parameters (Å^2^)

**Table:**

	*x*	*y*	*z*	*U*_iso_*/*U*_eq_	
C1	0.1596 (2)	0.38177 (9)	0.4486 (3)	0.0504 (5)	
C2	0.1301 (2)	0.33079 (11)	0.3050 (4)	0.0653 (6)	
H2	0.1735	0.2918	0.3386	0.078*	
C3	0.0355 (3)	0.33861 (14)	0.1113 (4)	0.0812 (8)	
H3	0.0168	0.3050	0.0117	0.097*	
C4	−0.0307 (3)	0.39525 (15)	0.0648 (4)	0.0864 (8)	
H4	−0.0953	0.3998	−0.0650	0.104*	
C5	−0.0026 (3)	0.44530 (13)	0.2077 (4)	0.0780 (7)	
H5	−0.0489	0.4836	0.1758	0.094*	
C6	0.0940 (2)	0.43918 (11)	0.3987 (4)	0.0617 (6)	
H6	0.1149	0.4737	0.4940	0.074*	
C7	0.4581 (2)	0.32309 (9)	0.4971 (4)	0.0563 (5)	
H7A	0.4214	0.2838	0.5369	0.068*	
H7B	0.4229	0.3295	0.3422	0.068*	
C8	0.6019 (2)	0.31741 (9)	0.5505 (3)	0.0536 (5)	
H8	0.6433	0.3027	0.6892	0.064*	
C9	0.6779 (2)	0.33075 (10)	0.4248 (3)	0.0549 (5)	
C10	0.8227 (3)	0.32188 (14)	0.5036 (4)	0.0874 (8)	
H10A	0.8445	0.3029	0.6456	0.131*	
H10B	0.8650	0.3629	0.5101	0.131*	
H10C	0.8513	0.2942	0.4057	0.131*	
C11	0.6331 (3)	0.35544 (14)	0.1992 (4)	0.0806 (8)	
H11A	0.5396	0.3548	0.1524	0.121*	
H11B	0.6672	0.3288	0.1051	0.121*	
H11C	0.6634	0.3987	0.1943	0.121*	
C12	0.45972 (18)	0.44109 (8)	0.5560 (3)	0.0407 (4)	
C13	0.41589 (19)	0.46298 (9)	0.3456 (3)	0.0475 (5)	
H13	0.3591	0.4380	0.2412	0.057*	
C14	0.54372 (19)	0.47809 (9)	0.7100 (3)	0.0471 (5)	
H14	0.5734	0.4633	0.8519	0.057*	
S1	0.28630 (5)	0.37450 (2)	0.68836 (8)	0.05203 (19)	
N1	0.42238 (16)	0.37861 (7)	0.6175 (2)	0.0466 (4)	
O1	0.28181 (15)	0.42921 (7)	0.8196 (2)	0.0655 (4)	
O2	0.28035 (15)	0.31145 (7)	0.7724 (3)	0.0727 (5)	

### Atomic displacement parameters (Å^2^)

**Table:**

	*U*^11^	*U*^22^	*U*^33^	*U*^12^	*U*^13^	*U*^23^
C1	0.0536 (12)	0.0418 (12)	0.0643 (12)	−0.0040 (10)	0.0305 (10)	−0.0043 (9)
C2	0.0595 (15)	0.0542 (14)	0.0857 (16)	−0.0051 (11)	0.0258 (13)	−0.0142 (12)
C3	0.0786 (19)	0.083 (2)	0.0821 (17)	−0.0127 (16)	0.0222 (15)	−0.0308 (15)
C4	0.0791 (19)	0.106 (2)	0.0718 (16)	0.0036 (17)	0.0157 (14)	−0.0058 (16)
C5	0.0846 (18)	0.0751 (18)	0.0774 (17)	0.0202 (15)	0.0273 (15)	0.0130 (14)
C6	0.0752 (16)	0.0511 (14)	0.0636 (13)	0.0042 (12)	0.0272 (12)	−0.0042 (10)
C7	0.0658 (15)	0.0336 (11)	0.0745 (13)	−0.0052 (10)	0.0277 (11)	−0.0031 (10)
C8	0.0649 (14)	0.0450 (12)	0.0524 (11)	0.0090 (10)	0.0182 (10)	0.0054 (9)
C9	0.0607 (14)	0.0512 (13)	0.0535 (11)	0.0067 (10)	0.0167 (10)	0.0007 (9)
C10	0.0680 (17)	0.116 (2)	0.0790 (16)	0.0122 (16)	0.0220 (14)	0.0009 (15)
C11	0.0872 (19)	0.096 (2)	0.0641 (14)	0.0167 (15)	0.0301 (14)	0.0185 (13)
C12	0.0507 (11)	0.0296 (9)	0.0451 (10)	−0.0018 (8)	0.0185 (9)	0.0006 (7)
C13	0.0579 (13)	0.0392 (11)	0.0440 (10)	−0.0108 (9)	0.0111 (9)	−0.0051 (8)
C14	0.0608 (13)	0.0413 (11)	0.0386 (9)	−0.0039 (10)	0.0123 (9)	0.0044 (8)
S1	0.0631 (4)	0.0435 (3)	0.0564 (3)	−0.0048 (3)	0.0282 (3)	0.0049 (2)
N1	0.0558 (10)	0.0311 (9)	0.0573 (9)	−0.0037 (7)	0.0225 (8)	0.0026 (7)
O1	0.0823 (11)	0.0655 (10)	0.0571 (8)	−0.0043 (8)	0.0330 (8)	−0.0125 (7)
O2	0.0808 (11)	0.0557 (10)	0.0903 (11)	−0.0069 (8)	0.0380 (9)	0.0261 (8)

### Geometric parameters (Å, °)

**Table:**

C1—C6	1.378 (3)	C9—C11	1.485 (3)
C1—C2	1.383 (3)	C9—C10	1.500 (3)
C1—S1	1.756 (2)	C10—H10A	0.9600
C2—C3	1.381 (3)	C10—H10B	0.9600
C2—H2	0.9300	C10—H10C	0.9600
C3—C4	1.364 (4)	C11—H11A	0.9600
C3—H3	0.9300	C11—H11B	0.9600
C4—C5	1.365 (4)	C11—H11C	0.9600
C4—H4	0.9300	C12—C14	1.374 (2)
C5—C6	1.375 (3)	C12—C13	1.379 (2)
C5—H5	0.9300	C12—N1	1.445 (2)
C6—H6	0.9300	C13—C14^i^	1.378 (2)
C7—C8	1.482 (3)	C13—H13	0.9300
C7—N1	1.494 (2)	C14—C13^i^	1.378 (2)
C7—H7A	0.9700	C14—H14	0.9300
C7—H7B	0.9700	S1—O1	1.4233 (14)
C8—C9	1.317 (3)	S1—O2	1.4257 (14)
C8—H8	0.9300	S1—N1	1.6345 (16)
			
C6—C1—C2	120.1 (2)	C9—C10—H10A	109.5
C6—C1—S1	120.00 (16)	C9—C10—H10B	109.5
C2—C1—S1	119.85 (17)	H10A—C10—H10B	109.5
C3—C2—C1	119.1 (2)	C9—C10—H10C	109.5
C3—C2—H2	120.5	H10A—C10—H10C	109.5
C1—C2—H2	120.5	H10B—C10—H10C	109.5
C4—C3—C2	120.5 (2)	C9—C11—H11A	109.5
C4—C3—H3	119.8	C9—C11—H11B	109.5
C2—C3—H3	119.8	H11A—C11—H11B	109.5
C3—C4—C5	120.4 (3)	C9—C11—H11C	109.5
C3—C4—H4	119.8	H11A—C11—H11C	109.5
C5—C4—H4	119.8	H11B—C11—H11C	109.5
C4—C5—C6	120.1 (2)	C14—C12—C13	119.93 (16)
C4—C5—H5	120.0	C14—C12—N1	118.85 (15)
C6—C5—H5	120.0	C13—C12—N1	121.18 (16)
C5—C6—C1	119.9 (2)	C14^i^—C13—C12	119.95 (17)
C5—C6—H6	120.1	C14^i^—C13—H13	120.0
C1—C6—H6	120.1	C12—C13—H13	120.0
C8—C7—N1	109.54 (16)	C12—C14—C13^i^	120.12 (16)
C8—C7—H7A	109.8	C12—C14—H14	119.9
N1—C7—H7A	109.8	C13^i^—C14—H14	119.9
C8—C7—H7B	109.8	O1—S1—O2	120.08 (9)
N1—C7—H7B	109.8	O1—S1—N1	107.20 (9)
H7A—C7—H7B	108.2	O2—S1—N1	106.63 (9)
C9—C8—C7	127.64 (19)	O1—S1—C1	107.84 (9)
C9—C8—H8	116.2	O2—S1—C1	107.89 (10)
C7—C8—H8	116.2	N1—S1—C1	106.46 (8)
C8—C9—C11	125.3 (2)	C12—N1—C7	115.31 (14)
C8—C9—C10	121.0 (2)	C12—N1—S1	116.94 (12)
C11—C9—C10	113.7 (2)	C7—N1—S1	118.15 (12)
			
C6—C1—C2—C3	0.7 (3)	C6—C1—S1—O2	143.23 (16)
S1—C1—C2—C3	−176.16 (17)	C2—C1—S1—O2	−39.90 (18)
C1—C2—C3—C4	−1.9 (4)	C6—C1—S1—N1	−102.63 (17)
C2—C3—C4—C5	1.1 (4)	C2—C1—S1—N1	74.23 (17)
C3—C4—C5—C6	0.8 (4)	C14—C12—N1—C7	−119.44 (19)
C4—C5—C6—C1	−2.0 (4)	C13—C12—N1—C7	58.5 (2)
C2—C1—C6—C5	1.2 (3)	C14—C12—N1—S1	94.90 (18)
S1—C1—C6—C5	178.08 (17)	C13—C12—N1—S1	−87.15 (19)
N1—C7—C8—C9	−108.2 (2)	C8—C7—N1—C12	65.6 (2)
C7—C8—C9—C11	0.7 (4)	C8—C7—N1—S1	−149.18 (14)
C7—C8—C9—C10	−179.6 (2)	O1—S1—N1—C12	−41.56 (15)
C14—C12—C13—C14^i^	−0.1 (3)	O2—S1—N1—C12	−171.36 (13)
N1—C12—C13—C14^i^	−177.99 (17)	C1—S1—N1—C12	73.64 (15)
C13—C12—C14—C13^i^	0.1 (3)	O1—S1—N1—C7	173.77 (14)
N1—C12—C14—C13^i^	178.04 (17)	O2—S1—N1—C7	43.97 (17)
C6—C1—S1—O1	12.14 (19)	C1—S1—N1—C7	−71.02 (16)
C2—C1—S1—O1	−170.99 (16)		

Symmetry codes: (i) −*x*+1, −*y*+1, −*z*+1.

### Hydrogen-bond geometry (Å, °)

**Table:**

*D*—H···*A*	*D*—H	H···*A*	*D*···*A*	*D*—H···*A*
C2—H2···O2^ii^	0.93	2.52	3.398 (3)	158

Symmetry codes: (ii) *x*, −*y*+1/2, *z*−1/2.
